# The Effect of Vaccination on the Evolution and Population Dynamics of Avian Paramyxovirus-1

**DOI:** 10.1371/journal.ppat.1000872

**Published:** 2010-04-22

**Authors:** Yee Ling Chong, Abinash Padhi, Peter J. Hudson, Mary Poss

**Affiliations:** 1 Center for Infectious Disease Dynamics, Department of Biology, The Pennsylvania State University, University Park, Pennsylvania, United States of America; 2 Fogarty International Center, National Institutes of Health, Bethesda, Maryland, United States of America; Centro de Biología Molecular Severo Ochoa (CSIC-UAM), Spain

## Abstract

Newcastle Disease Virus (NDV) is a pathogenic strain of avian paramyxovirus (aPMV-1) that is among the most serious of disease threats to the poultry industry worldwide. Viral diversity is high in aPMV-1; eight genotypes are recognized based on phylogenetic reconstruction of gene sequences. Modified live vaccines have been developed to decrease the economic losses caused by this virus. Vaccines derived from avirulent genotype II strains were developed in the 1950s and are in use globally, whereas Australian strains belonging to genotype I were developed as vaccines in the 1970s and are used mainly in Asia. In this study, we evaluated the consequences of attenuated live virus vaccination on the evolution of aPMV-1 genotypes. There was phylogenetic incongruence among trees based on individual genes and complete coding region of 54 full length aPMV-1 genomes, suggesting that recombinant sequences were present in the data set. Subsequently, five recombinant genomes were identified, four of which contained sequences from either genotype I or II. The population history of vaccine-related genotype II strains was distinct from other aPMV-1 genotypes; genotype II emerged in the late 19^th^ century and is evolving more slowly than other genotypes, which emerged in the 1960s. Despite vaccination efforts, genotype II viruses have experienced constant population growth to the present. In contrast, other contemporary genotypes showed population declines in the late 1990s. Additionally, genotype I and II viruses, which are circulating in the presence of homotypic vaccine pressure, have unique selection profiles compared to nonvaccine-related strains. Collectively, these data show that vaccination with live attenuated viruses has changed the evolution of aPMV-1 by maintaining a large effective population size of a vaccine-related genotype, allowing for coinfection and recombination of vaccine and wild type strains, and by applying unique selective pressures on viral glycoproteins.

## Author Summary

Modified live virus (MLV) vaccines have been effective in reducing disease burden and economic loss caused by Newcastle Disease (ND) in domestic poultry. Because the vaccine is a live virus, it is transmissible among birds. Thus, vaccination strategies have the potential to impact the evolutionary genetics of wild type strains of aPMV-1 including those that cause ND. In this report, we provided evidence that viruses isolated from wild and domestic birds have recombined with vaccine strains, because vaccinated birds are protected from disease but not infection with other strains of aPMV-1. Despite the use of vaccines since the 1950s, the population size of the strain from which the most widely used vaccine was derived has steadily increased. In contrast, other contemporary genotypes, which emerged in the 1960s, experienced a decline in population size in 1998, which may reflect a change in poultry farming practices or disease. Vaccination imposed a unique selection profile on the genotypes derived from the vaccine-related strains when compared with nonvaccine-related strains. Although modified live viruses are important for controlling Newcastle Disease, the potential of vaccination strategies to change viral diversity and population dynamics should be considered.

## Introduction

Live attenuated virus vaccines have been successfully employed in veterinary medicine to prevent the economic impact of many diseases in poultry and livestock. However, the role of vaccination with attenuated viruses on the evolution of wild type strains is not often considered. Antigenic escape because of strong selection by vaccines, emergence of new strains through recombination, and increased virulence to expedite transmission of new genotypes in vaccinated populations are of potential concern. In this paper, we explored the consequences of vaccination on the evolution of class II aPMV-1, which is the etiological agent of ND.

NDV, a single-stranded, non-segmented, negative-sense RNA virus of the genus *Avulavirus*, family Paramyxoviridae, infects a wide range of domestic and wild bird species worldwide, and causes a significant economic burden to the poultry industry [1]. The first outbreaks of NDV were reported during the mid 1920s in Java, Indonesia and Newcastle-upon-Tyne, England [2], and within a few years were occurring throughout the world [3]. The name ND is reserved exclusively for the disease that results from infection with strains of aPMV-1 that are pathogenic for domestic chickens [4]. aPMV-1 has been grouped by virulence phenotype, with lentogenic, mesogenic, and velogenic strains representing increasing levels of virulence, ranging from subclinical infections with moderate respiratory involvement to extensive hemorrhagic lesions and neurological signs [5]. Inactivated vaccines were first made commercially available to the poultry industry in 1946, but because they provided incomplete protection against ND [6], they were replaced with live lentogenic NDV vaccines. Although these vaccines reduce disease, they do not always prevent infection and birds can shed both vaccine and challenge strains of the virus [7], [8], [9].

aPMV-1 genome size is approximately 15 kb and encodes six genes, which produce nucleocapsid protein (NP), phosphoprotein (P), matrix protein (M), fusion protein (F), hemagglutinin-neuraminidase (HN), and polymerase protein (L) [10], [11]. RNA-editing of P gene creates two additional proteins, V and W [12]. There are nine serotypes of aPMV-1; viruses associated with ND are in serogroup 1. Within serogroup 1 there are 2 major subdivisions, class I and II, based on phylogenetic grouping of the F gene [13]. Class I aPMV-1 are primarily recovered from waterfowl or samples from U.S. live bird markets, while the isolates from class II are commonly derived from poultry and other avian species [5], [14]. Eight genotypes of class II aPMV-1 can be identified [13]. Viruses belonging to genotypes I-IV have circulated since the 1930's. Genotype I and II consist of both lentogenic and velogenic viruses and have been associated with ND outbreaks in Australia and North America, respectively [5], [15], [16]. These viruses have been attenuated in culture and are used as modified live vaccines [16]. Genotypes V-VIII were first recognized in the mid-1960s [13] and contained only virulent viruses [16]. Genotype V was responsible for the second panzootic of ND in Europe from 1970–1974 and has been detected sporadically thereafter [17]. Genotype VI was described mainly from the Middle East and Asia during the 1980's–1990's [18] and genotype VII and VIII were reported in the 1990's from several countries [19], [20], [21], [22], [23], [24]. All genotypes, except IV [16], are still in circulation.

RNA viruses typically have a high mutation rate due to low fidelity and processivity of their polymerase [25], which coupled with a high replication rate and short generation time [26] lead to high evolutionary rates. In addition, evidence is accumulating that recombination is an important process driving genotype diversity for many RNA viruses [27], [28], [29]. Although recombination was not thought to contribute to aPMV-1 evolution [30], [31], evidence of recombination in NDV has recently been reported [32], [33], [34]. This debate may be due, in part, to the reliance on a single gene for determining virus diversity and phylogeny. Because recombination can lead to the emergence of novel virus strains of unknown virulence [35], [36], [37], [38], a better understanding of the role of recombination in circulating aPMV-1 is warranted.

In this study, we explored how vaccination strategies in poultry farming have shaped the evolution of this important avian virus using complete genome sequences available in GenBank. Specifically our objectives were to 1) determine if recombination was evident among full length class II aPMV-1; 2) estimate evolutionary rates of each genotype; 3) estimate the effective population size of each genotype; and 4) determine the selective forces on vaccine-related and nonvaccine-related wild type genotypes. Our results confirm that recombination is an important process in this negative sense RNA virus and that vaccine-related strains have an evolutionary history that is unique from nonvaccine-related strains, which includes distinct evolutionary rates, temporal changes in population size, and selection profiles.

## Results

### Phylogenomic analyses

**Figure 1:**
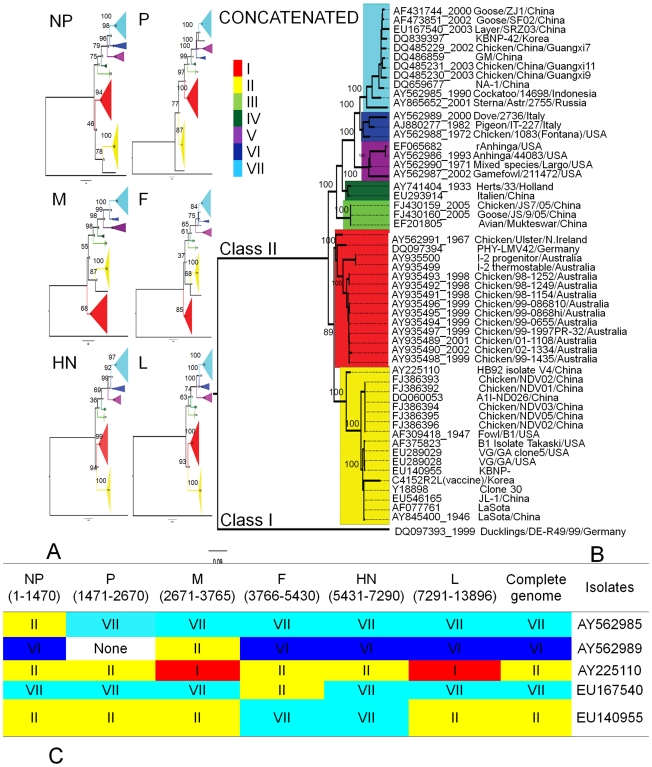
Maximum likelihood trees showing phylogenetic relationships among different genotypes. A total of 54 isolates representing seven different genotypes were used in the phylogenetic reconstruction. (**A**) Genotype topology based on each of the six aPMV-1 genes. Area of the triangle is proportional to the number of isolates within the genotype. (**B**) ML tree inferred from the concatenated gene sequences of the 54 isolates. Genbank accession number is shown for each isolate. Bootstrap supports are given at the base of the nodes. All trees are rooted with strain DQ097393, which belongs to class I aPMV-1. The trees are midpoint rooted for clarity. (**C**) Diagram of genotype affiliation of each gene for isolates that showed topological incongruence in different gene trees. Genotypes I, II, III, IV, V, VI, and VII, are coded with red, yellow, lime-green, dark-green, purple, cyan, and blue colors, respectively.

The current phylogenetic classification of aPMV-1 strains is based on either full or partial nucleotide sequence of the M, F, or L genes. To determine if all genes in the viral genome provided consistent phylogenetic profiles, we obtained 54 full length class II aPMV-1 sequences from Genbank and generated nucleotide data sets for each of the six genes and a concatenated sequence of all protein coding regions. Maximum likelihood (ML) phylogenies were reconstructed for all sequence data sets under the appropriate nucleotide substitution model selected for each data set (Figure 1). Each gene tree and the concatenated tree revealed seven distinct genotypes within the class II aPMV-1. However, genotype affiliations were not congruent among different genes (Figure 1A). While genotype III, IV, V, VI, and VII were monophyletic, genotype I and II showed inconsistent phylogenetic relationships (Figure 1A and B). Three distinct patterns of affiliations of genotype I and II were observed among different gene trees. The NP, HN, L, and concatenated gene trees consistently placed genotypes I and II in a sister clade to other genotypes. In the P gene tree, genotype II formed a basal clade while genotype I clustered with the remaining genotypes and in the M and F gene trees, genotype I was the basal clade and genotype II clustered with the remaining genotypes.

Several taxa changed genotype affiliations in different gene trees and all discordant taxa were affiliated with vaccine-related genotype II in some gene trees (Figure 1C). For example, isolate AY562985 (Cockatoo/14698/Indonesia/1990) was affiliated with genotype II in the NP gene tree but with genotype VII in all other gene trees. This isolate occupied a long branch in the genotype VII sequences in the P gene tree. Isolate AY562989 (Dove/2736/Italy/2000) was affiliated with genotype II in the M gene tree but with genotype VI in all remaining trees except the P gene, in which it was an outlier to all other genotypes. Isolate AY225110 (HB92 isolate V4 vaccine/China) affiliated with genotype I in M and L gene trees but with genotype II in other gene trees. Isolate EU167540 (Layer/SRZ03/China/2003) affiliated with genotype VII in all gene trees but it occupied a long branch in genotype II in F gene tree. A Shimodaira-Hasegawa test (SH-test) provided statistical support of taxon incongruence (p<0.005) among the gene trees (data not shown). Phylogenetic incongruence among genes suggests that recombination might play a role in class II aPMV-1 diversity.

### Detection of recombinant viruses

To further investigate the possibility of recombination among the full length aPMV-1 sequences, we used seven different algorithms implemented in the RDP3 program [39], [40]. Chimeric NDV vaccine strain EU140955, which has the genotype II La Sota vaccine strain backbone and the F and HN genes from a contemporary genotype VII virus (Figure 1C), was included as a control to evaluate the prediction capability of the program. The predicted recombination breakpoint (detected by five methods with p-value<10^−5^) at position 7119 of the concatenated EU140955 matched correctly with the end of a *Spe*I restriction site of this chimeric strain where HN sequences were inserted from the KBNP-4152 strain. The *Mlu*I restriction site used to generate the chimera is within the intergenic region between M and F genes and is not present in our sequences. However, the RDP3 program reasonably identified the 5′ breakpoint at the 8th nucleotide of the F-gene. Two additional recombination breakpoints within this insert were also detected by the GENECONV and Bootscan methods (p-value 2.29×10^−3^ and 3.35×10^−2^). These corresponded to the positions in the F gene segment of KBNP-4152 strain that were mutagenized to attenuate recombinant strain EU140955. Thus, we conclude that the RDP3 program accurately identifies recombination if five or more methods have statistical support of p*≤*10^−5^ for the breakpoints and we propose that any breakpoints statistically supported with only one or two methods should be carefully interpreted.

**Figure 2:**
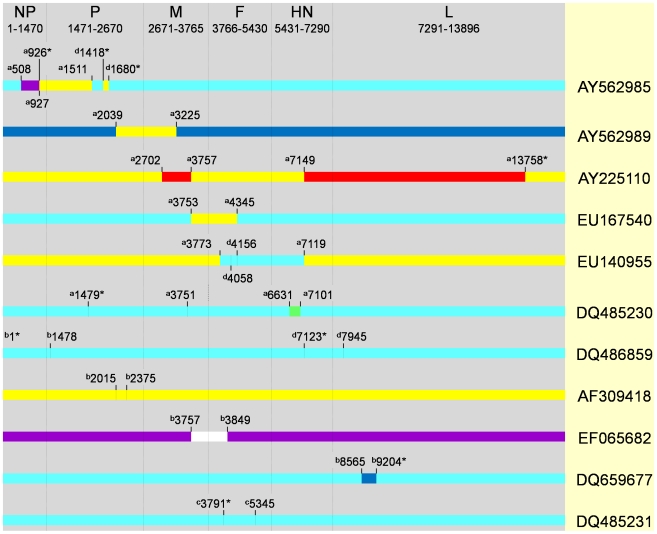
Recombination breakpoint map of eleven isolates detected by different methods implemented in RDP3 program. The nucleotide position of breakpoints in the concatenated genome is indicated. Regions that correspond to different genotypes are shown using the color scheme described in Figure 1. Superscripts a and b indicate statistical support (p-value≤10^−5^) from 5 or 3–4 methods, respectively. Superscripts c and d indicate statistical support (p-value≤10^−3^) from 2 to 4 or 1 method, respectively. Asterisk indicates that breakpoints could not be identified. Genes are not drawn to scale.

**Figure 3:**
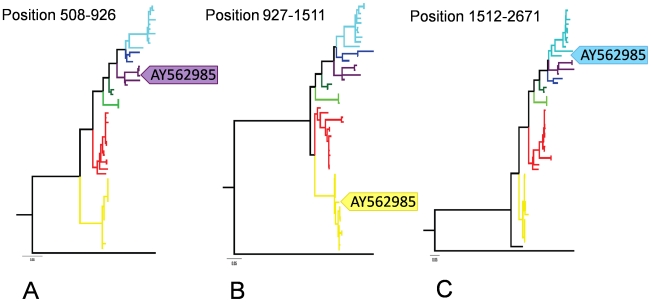
ML trees on the three putative recombinant regions identified in AY562985. A–C represent the phylogenies of regions spanning nucleotide position 508–926, 927–1511, and 1512–2671, respectively. The position of AY562985 is highlighted in each tree. Genotypes I, II, III, V, VI, and VII are coded using the color scheme described in Figure 1. Trees are rooted with strain DQ097393, which belongs to class I aPMV-1.

Using the stringent criteria defined above, a total of five putative recombinant isolates were detected (Figure 2). Four of these isolates, AY562985, EU167540, AY562989, AY225110, were those that showed discordant phylogenies described above (Figure 1C), and in each case, some recombinant regions were derived from genotype II sequences. Isolate AY562985 is predominantly genotype VII and had evidence of two recombination events based on RDP3 predictions. We confirmed that regions 508 to 926 (in NP) and 927 to 1511 (NP and P) are related to genotypes V and II, respectively by partitioning the data sets at the predicted break points and reconstructing a ML phylogeny (Figure 3). In the region 927 to 1511, AY562985 had seven unique synonymous substitution sites compared to the other sixteen genotype II sequences in our dataset. Isolate AY562989, which is predominantly of genotype VI origin, also contained a putative recombinant region spanning positions 2039 to 3225, which was derived from genotype II and had three unique non-synonymous substitution sites compared to other genotype II sequences. Isolate AY225110 is a chimera of genotype I and II sequences. Compared to genotype II sequences, there were seven unique sites, four of which were non-synonymous substitutions, from the 5′ end of NP to position 2702; two synonymous and two non-synonymous substitution sites in the 3757–7149 fragment; and two synonymous and seven non-synonymous substitution sites within the region from 13758 to the 3′ end of L. For isolate EU167540, a putative recombination region between position 3753 and 4345 was affiliated with genotype II and had one non-synonymous substitution site compared to other strains. Thus, genotype II, which is used in vaccines globally, has recombined with at least three other class II aPMV-1 strains and the recombinant viruses have been isolated from both domestic and wild birds.

The fifth virus identified by all RDP3 methods, DQ485230, was a genotype VII isolate that contained a small region within the HN gene contributed by genotype III. In addition, a region spanning 1479 to 3751 in P and M appeared to be derived from a different genotype VII virus. Inter-genotype recombination was also detected by fewer than five of the RDP3 methods in DQ486859(GM/China), DQ485231(Guangxi11/China/2003) and AF309418(Fowl/B1/USA/1947). Genotype VII isolate DQ659677(NA-1/China) contained a 640 bp region within L contributed from genotype VI. The origin of the putative recombinant fragment spanning the M and F genes of EF065682(rAnhinga/USA) could not be determined. These data provide compelling evidence that genotypes II and VII are most commonly associated with recombinant viruses and that both intra- and intergenic recombination events can be detected using full genome sequence analysis.

### Phylogenomic analyses without recombinant sequences

**Figure 4:**
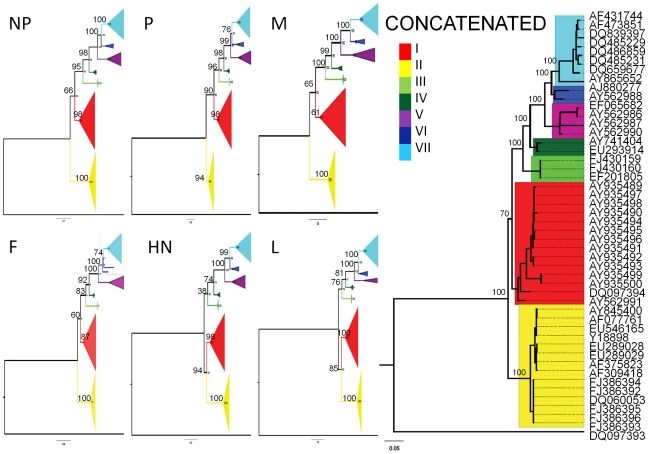
Phylogenetic relationships among 48 non-recombinant isolates. A maximum likelihood tree was produced after removal of the six putative recombinant sequences. The color scheme, rooting, and bootstrap values are as described in Figure 1.

Phylogenies of individual and concatenated genes were reconstructed after the removal of the five putative recombinant isolates and chimeric vaccine strain EU140955 (Figure 4). Consistent with Figure 1A and 1B, genotypes III - VII clustered together as a monophyletic group in all trees. All taxa were consistently affiliated with a single genotype and there were no long branches associated with any genotype. Two of the three original patterns of phylogenetic affiliation were retained following removal of recombinant sequences. The placement of genotypes based on HN and L was the same with or without recombinants; genotype I and II were sister groups to genotype III, IV, V, VI, and VII (Figure 1A; Figure 4). All of the remaining trees presented a topology similar to that of the P gene-tree before recombinant removal, which placed genotype I with III-VII. It is noteworthy that in the absence of recombinant sequences, genotype II is never clustered with genotypes III-VII, as was seen with trees based on M and F in the presence of recombinant sequences (Figure 1A).

### Evolutionary rates and population dynamics

**Figure 5:**
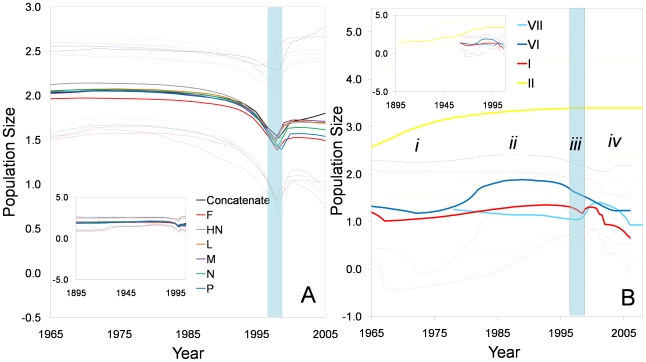
Bayesian skyline plots (BSP) depicting effective population size of class II aPMV-1 over time. Effective population size estimates are expressed on a logarithmic scale (*y* axis). Solid lines denote the median estimates and the dotted lines give the 95% HPD intervals of the estimates. The shaded light blue region indicates a population bottleneck. BSP based on (**A**) individual genes and concatenated genome sequences. The figure highlights the interval from 1965–2005; the entire estimate is displayed in the inset. The dark blue, red, pink, blue, light green, light blue and green lines are the estimates based on the concatenated sequence, NP, P, M, F, HN and L genes, respectively; (**B**) individual genotypes (I, II, VI, and VII) using complete F gene sequence. Symbols *i-iv* represent four phases of aPMV-1 history (described in detail in text). Genotype I, II, VI and VII, are coded by red, yellow, dark blue, and light blue lines, respectively.

We inferred the evolutionary rates and past population dynamics of class II aPMV-1 using a Bayesian coalescent approach [41]. This analysis was based on all full length genome sequences in the data set which had a date of isolation and excluded the six recombinant sequences. Bayesian estimates of the evolutionary rates of each gene and concatenated coding genome of class II aPMV-1 were between 0.98×10^−3^–1.56×10^−3^ substitutions/site/year (Table 1). Evolutionary rate estimates under a relaxed clock with HKY+G_4_ (Table S4) and GTR+G_6_ (Table 1) substitution models were consistent. The time to the most recent common ancestor (TMRCA) of class II aPMV-1 was estimated to be between 114 and 137 years before 2005, or between year 1868 and 1891. Bayesian skyline plots (BSP) were used to infer how effective population size has changed with time [41], [42]. All six protein-coding genes and the concatenated genome maintained constant effective population size until the late 1990's (Figure 5A). In 1997-8 there was an abrupt decline in the population with recovery from this event in the early 2000.

**Table 1:** Bayesian estimates of evolutionary rates (nucleotide substitutions per site per year) and TMRCAs (in year) for different genes of class II PMV-1.

Gene	Evolutionary rate (×10^−3^ sub/site/yr)	TMRCA in year (95% HPD)
NP	0.98 (0.45–1.50)	1868 (1713–1927)
P	1.56 (0.78–2.32)	1884 (1750–1931)
M	1.17 (0.54–1.76)	1872 (1737–1931)
F	1.35 (0.71–1.98)	1891 (1777–1931)
HN	1.10 (0.51–1.68)	1869 (1719–1928)
L	1.02 (0.59–1.44)	1878 (1764–1924)
Concatenate	1.19 (0.50–1.74)	1885 (1731–1929)

Estimates are based on the relaxed clock with uncorrelated exponential distribution (UCED).

To determine if all genotypes exhibited the same population history observed for the composite genotype data set, we repeated the analysis based on 97 dated full length F genes, which provided a larger data set for this analysis. Isolates from genotype III, IV, and V were not included because limited numbers of dated sequences were available. The TMRCA for genotype II was estimated to be 1899±20 years, and the estimated evolutionary rates were between 0.3–1.1×10^−4^ substitutions/site/year, making this the slowest evolving aPMV-1 genotype (Table 2). Genotype I and VI emerged in the early 1960's and had higher evolutionary rates than genotype II. The most recently emerged strain was genotype VII, which dates to the late 1970's and had the highest evolutionary rate. BSP analyses based on the F gene demonstrated that each genotype had a unique population history. Prior to the emergence of genotype VII in the 1970s (phase i), genotype II showed an increase in population size (Figure 5B). After the emergence of genotype VII (phase ii) the population size of genotype VI began to increase, while that of genotypes I and VII were relatively constant. Phase iii depicts the time of the population bottleneck observed in Figure 5A, which was based on all genes in the composite genotype data set. Only genotypes I and VI show a trend for decreasing population size during this time. The last decade has been the most dynamical for the four genotypes of aPMV-1 (phase iv; Figure 5B). Genotypes I and VII showed a marginal increase in effective population size followed by a decline; genotype I has continued to decline, whereas genotype VII appears to have stabilized. Genotype VI showed continuous decline in population size in phase iv (Figure 5B). Although estimates of population sizes for genotypes I, VI, and VII, have some degree of overlaps at the 95% posterior limit, genotype II shows no sign of reduction in effective population size since its origin (Figure 5B).

**Table 2:** Bayesian estimates of evolutionary rates (nucleotide substitutions per site per year) and TMRCAs (in year) based on the complete F gene of class II PMV-1 genotypes.

Genotype	Evolutionary rate (×10^-3^ sub/site/yr)	TMRCA in year (95% HPD)
I	1.73 (0.59–2.82)	1964 (1928–1969)
II	0.06 (0.03–0.11)	1899 (1888–1919)
VI	1.80 (0.85–2.69)	1963 (1915–1974)
VII	2.89 (1.27–4.43)	1978 (1940–1989)

Estimates are based on the relaxed clock with uncorrelated exponential distribution (UCED).

### Selection profiles

We compared the selection profiles on protein coding genes of genotypes I and II, which include strains that are circulating in the face of homotypic vaccination pressure (designated as the vaccine-related group), and genotypes III-VII (designated as the nonvaccine-related strains) (Table 3). Overall, the global rate of non-synonymous to synonymous substitutions (dN/dS) for all protein coding genes were less than 1, indicating purifying selection has been the major driving force in the evolution of class II aPMV-1 viruses. There were no codons identified to be under positive selection in M and L genes in either group (Table 3). However, there was a clear difference in the codon based selection profiles of N, P, F and HN genes between the vaccine- and nonvaccine-related groups. In the vaccine-related group, only the surface protein encoding genes, F and HN, had positively selected codons. Both groups had a single site identified in F; these were at codon 115 within the F_0_ cleavage site for the vaccine-related group and codon 28 in the signal peptide for the nonvaccine-related group. There were 3 positively selected sites identified in the HN gene in the vaccine-related group and one in the non vaccine-related group. In contrast, the P gene of nonvaccine-related genotypes III-VII had three sites predicted to be under positive selection but no sites were identified in the vaccine-related group. Thus, selection is focused on HN in vaccine-related groups and on P in nonvaccine-related genotypes and there are no shared sites under positive selection between the two groups.

**Table 3:** Site**-**specific selection analysis for each coding gene of vaccine- and nonvaccine-related groups.

Gene	Total number of codons	Global dN/dS*(codon position¶)	
		Vaccine-related group	Nonvaccine-related group
NP	489	0.119 (None)	0.100 (467§)
P	395	0.266 (None)	0.336 (87§, 90§, 380§)
M	364	0.176 (None)	0.123 (None)
F	553	0.166 (115¥)	0.139 (28†)
HN	616	0.166 (266§, 495†, 522†)	0.171 (508§)
L	2204	0.088 (None)	0.094 (None)

Strains used for selection analyses in both groups are mentioned in Table S1.

**¶:** Position of codon under positive selection with p<0.05.

**¥:** Significant in PAML analysis.

**§:** Significant in FEL analysis.

**†:** Significant in both FEL and PAML analysis.

*: Rate of Non-synonymous (dN) to Synonymous (dS) substitution.

## Discussion

Our study explored the forces shaping the evolutionary history of class II aPMV-1 using available full genome sequences. We demonstrated that genotype affiliations based on individual genes and concatenated full length genomes of class II aPMV-1 were not consistent. This may account for discrepancies reported for genotype groupings that are based on partial or complete sequences of a single gene [13], [18], [43]. Topological incongruence among the gene trees reflects different evolutionary histories of each gene [44]; recombination is the most plausible explanation for this. The role of recombination in the evolution of aPMV-1, and negative sense RNA viruses in general, has been debated. For example, Sakaguchi et al. [30] and Toyoda et al, [31] reported consistent topological placement of different NDV strains in both F and HN gene trees. Seal et al. [45] also reported that there was no evidence of recombination among NDV M gene sequences. Although recombination is more common in positive-sense RNA viruses and can be explained by several genetic mechanisms [46], there is increasing evidence of homologous recombination in several non-segmented negative-sense RNA viruses [32], [47], [48], [49], [50]. Our approach differs from those used in previous studies of aPMV-1 evolution because we evaluated full genome sequences and tested individual recombinant regions with phylogeny-based incongruence tests. Thus, we show that all class II aPMV-1 genes have evidence of recombination breakpoints, that multiple recombination events are discernable in some isolates, and that both intragenic and intergenic recombination events are evident. We considered the possibility that the recombinants detected in our analysis were the result of laboratory artifacts, as has been previously suggested [32], [51]. Laboratory contamination is of concern because vaccine derived strains contributed the majority of the recombinant regions and these strains might have been present in laboratories sequencing field aPMV-1 isolates. The presence of unique nucleotide substitutions in the recombinant regions compared to the comparable region of the predicted parental genotypes suggests that these regions did not arise due to contamination with vaccine strains deposited in the sequence databases.

Our identification of recombinants derived from vaccine strains indicates that birds can be simultaneously infected with the live virus vaccine and other circulating aPMV-1 genotypes. Indeed vaccination is reported to protect poultry from disease but not always from infection with other strains [7], [8], [9]. Suboptimal vaccination strategies could also lead to birds becoming infected with both a vaccine strain and circulating genotype, which can alter viral virulence [16]. This is an important issue for poultry management. Lack of vaccine efficacy has not frequently been reported in the United States but other countries such as Nigeria [52], Korea [53], Taiwan [54], and China [34], [55] have experienced vaccine failures. Recombination between wild type virus and vaccine strains is not unique to aPMV-1; vaccine recombinants of bovine viral diarrhea virus (associated with fatal mucosal disease) [35], poliovirus (associated with paralytic poliomyelitis) [36], [37]; and infectious bursal disease virus [38] have all been reported. This raises concerns that modified live virus vaccines, although efficacious, may facilitate emergences of new strains with unpredictable phenotypes through recombination with circulating viruses.

The evolutionary rates presented here for class II aPMV-1 are compatible with the rates estimated for other RNA viruses (e.g. [56], [57], [58]), suggesting that class II aPMV-1 is also a rapidly evolving RNA virus. The relatively lower evolutionary rate for genotype II is consistent with the rate that was previously reported for avirulent NDV [33]. The larger effective population size, which counters the impact of genetic drift, is a possible explanation for lower evolutionary rates of genotype II. Based on these rate estimates, the TMRCA of this virus is estimated to be between 1868–1891, which is earlier than the first recorded outbreak of ND in Indonesia and England in the 1920's [2]. However, our data are in line with observations by Macpherson [59], who suggested that an outbreak of disease in domestic birds that occurred in Northwest Scotland from 1897–1898 was due to NDV.

The demographic history of class II aPMV-1 determined by Bayesian skyline plots indicated that there was an abrupt decline in population size during 1997–98. Although the factors responsible for such an abrupt decline in class II aPMV-1 are not known, the impact of a severe El-Nino event during that time frame [60] or the slaughter of millions of domestic fowl during the first outbreak of H5N1 avian influenza virus in 1997 [61], [62] could be possible explanations.

In contrast to the other genotypes, genotype II was not impacted by factors causing population decline in the late 1990s. We expected that vaccination, which started worldwide in the 1950s, should have limited the number of susceptible avian hosts, thus causing a bottleneck for this genotype. However, the impact of NDV vaccination is not seen in the BSP. It is possible that the data available in GenBank is insufficient to capture the population history of this genotype. However, a plausible explanation for the absence of a population bottleneck could be that genotype II NDV is maintained as an asymptomatic infection because it is continually introduced to susceptible populations as a modified live vaccine. Vaccination effectively prevents birds from developing disease when exposed to a virulent strain, but does not prevent shedding [7]. Thus, the number of available susceptible hosts may not dictate genotype II population demographics.

Although overall all genes of class II aPMV-1 are evolving under purifying selection consistent with other paramyxoviruses [33], [56], [63], distinctive profiles of positively selected codons were shown in both vaccine- and nonvaccine-related groups. Notably, P gene had the highest dN/dS ratio of any gene and had three sites predicted to be under selection in the nonvaccine-related group; there were no sites under selection in P gene in the vaccine related group. In contrast, HN had three sites predicted to be under selection in the vaccine related group. Of interest, codon 115 in the F cleavage domain is positively selected only in vaccine-related groups. Previous studies have reported that a single amino acid substitution at codon-115, which falls within the F0 cleavage site, resulted in a dramatic change from an avirulent infection to highly virulent NDV [64], [65], [66]. In contrast to the results reported in the present study, Miller et al [33] did not identify codon115 in F gene under positive selection. This is likely due to differences in the data sets because factors such as sequence length, sequence divergence, and the number of sequences can determine the ability to detect positively selected sites [67]. Thus, the vaccine-related genotypes I and II maintain a phenotypic mixture of strains with different infection and pathogenic potential and selection profiles.

## Materials and Methods

### Sequences data collection and phylogenetic analyses

A total of 54 complete genome sequences of class II aPMV-1 representing different avian hosts, geographic regions, year of isolation, and genotypes (based on previous published phylogenetic grouping) were retrieved from GenBank. The coding genome sequences were aligned using MEGA version 4 [68]. Six separate coding gene sequences datasets (for NP, P, M, F, HN and L genes) were generated (see Table S1) and a concatenated genome sequence from these six coding gene sequences was generated using Mesquite version 1.12 (http://mesquiteproject.org). Appropriate model of nucleotide substitution for each dataset was selected by the hierarchical likelihood ratio test implemented in Modeltest version 3.7 [69]. Maximum likelihood (ML) trees were reconstructed for all data sets using the heuristic search option, implementing stepwise addition with 100 random addition replicates and tree bisection-reconnection branch swapping in PAUP* version 4beta10 [70] and PHYML 3.412 [71] with 100 non-parametric bootstrapping replicates analyses. The inferred trees were visualized with FigTree version 1.12 (http://tree.bio.ed.ac.uk/software/figtree/) and the congruency of topology placement of class II aPMV-1 genotypes based on each gene and concatenated genome was tested using the Shimadoira, Hasegawa (SH) test [72] implemented in PAUP. The concatenated tree was constrained and tested versus other gene trees.

### Putative recombination detection

The recombination predictions of the concatenated genome sequences were conducted with a suite of programs within the RDP3 package [39], [40]. The individual programs RDP [39], GENECONV [73], Bootscan [40], Maximum Chi [74], Chimaera [75], SiScan [76] and 3Seq [77], were implemented for the analysis. Since no single program provided optimal performance under all conditions, any event supported by five or more methods with p-values ≤10^−5^ was the criteria used for positive recombination breakpoints identification. The breakpoint position and the putative parental sequences were also determined.

### Estimation of evolutionary rates and past population dynamics

Twenty six full length genome sequences for which year of isolation was available were used to infer evolutionary rate and dates using BEAST version 1.4.8 [41]. Demographic history of a population/species using multi-locus data, even from a small number of individuals, can precisely recover past bottlenecks in population size that cannot be characterized by analysis of a single locus [78]. Given this fact, estimates based on the whole genome sequence data are expected to be more reliable. To determine the population history of individual genotypes, 149 complete F gene sequences, which had dates of collection, were retrieved from GenBank. Phylogenetic analyses were done as described previously (Table S2). From the ML tree, a total of 97 isolates from genotype I, II, VI, and VII were selected to infer evolutionary rates and population dynamics. These included all available dated full length sequences from Genotypes I (24 sequences), II (28 sequences), and VI (23 sequences). The majority (95%) of genotype VII sequences were of Chinese origin. Thus, we included the seven non-Chinese sequences and picked an additional 15 sequences based on phylogenetic diversity to represent genotype VII in our analyses. The evolutionary rate (nucleotide substitutions per site per year) of each gene and concatenated genome was estimated using the Bayesian Markov chain Monte Carlo analyses (independent assumption of codivergence). Substitution models of both HKY + G_4_ and GTR + G_6_ with estimated base frequencies, gamma and invariable site portion were used, with uncertainty in the data reflected in the 95% high-probability density (HPD) intervals. Strict clock and uncorrelated exponential (UCED) relaxed clock models were attempted independently, and the best-fit clock model was determined to be UCED based on the Bayes Factor calculated from their posterior distributions (Table S3). The Coalescent Bayesian skyline plot (BSP) was used to infer the past population dynamics. The BSP was constructed using the growth rate and demographic parameters from the selected best-fit models. Bayesian Markov Chain Monte Carlo (BMCMC) analyses were run for 5–10×10^8^ generations depending on each dataset. Convergence of trees was checked using Tracer v1.4.1 (http://beast.bio.ed.ac.uk/Tracer).

### Selection analysis

Selection analyses were done based on datasets without putative recombinant sequences because recombination can result in falsely identifying positive selection [79]. The datasets were split into a vaccine-related group, which included strains from genotypes I and II, and nonvaccine-related group, which included strains from genotypes III, IV, V, VI, VII (see Table S2). Positively selected codons were detected using Fixed- Effect Likelihood (FEL) via the Datamonkey website (http://www.datamonkey.org/) and ML approach implemented in CODEML (PAML package version 3.15)[80]. For FEL analysis, p-values less than 0.05 were used to support positive selection. For PAML analysis, the likelihood ratio test was used to compare M1a, M7 and M8a models that assume no positive selection (*ω<1*) with those M2a and M8 models that assume positive selection (*ω>1*)[81].

## Supporting Information

Table S1 Full length genome sequence of class II aPMV-1 used in this study
(0.13 MB DOC)

Table S2 Full length F gene sequences used in BEAST analyses.
(0.15 MB DOC)

Table S3 The evolutionary rates and time to the most recent common ancestor (TMRCA) of each gene and concatenated genome based on GTR substitution model.
(0.06 MB DOC)

Table S4 The evolutionary rates and time to the most recent common ancestor (TMRCA) of each gene and concatenated genome based on HKY substitution model, using both strict and relaxed (Uncorrelated Exponential) clock model.
(0.06 MB DOC)
