# Supplementary material for: The Effect of Vaccination on the Evolution and Population Dynamics of Avian Paramyxovirus-1
Source: PLoS Pathog. 2010 Apr 22;6(4):e1000872. doi: 10.1371/journal.ppat.1000872 (PMC2858710; doi:10.1371/journal.ppat.1000872)
Supplement: Table S1 — Full length genome sequence of class II aPMV-1 used in this study (0.13 MB DOC) [file ppat.1000872.s001.doc]

**Table S1.** Full length genome sequence of class II aPMV-1 used in this study.

| GenBank accession number | Genotype | Strain/isolation name | Year | Country | Host |
| --- | --- | --- | --- | --- | --- |
| AF077761‡ | II | LaSota | N.A. | N.A. | N.A. |
| AF309418‡ | II | B1 | 1947 | USA | N.A. |
| AF375823‡ | II | strain B1 isolate Takaski | N.A. | USA | N.A. |
| AF431744* | VII | ZJ1 | 2000 | China | goose |
| AF473851* | VII | SF02 | 2002 | China | goose |
| AJ880277* | VI | IT-227/82 | 1982 | Italy | pigeon |
| AY225110 | II | strain HB92 isolate V4 | N.A. | China | N.A. |
| AY562985 | VII | cockatoo/Indonesia/14698/90 | 1990 | Indonesia | cockatoo |
| AY562986* | V | anhinga/U.S.(Fl)/44083/93 | 1993 | USA; Florida | anhinga |
| AY562987* | V | gamefowl/U.S.(CA)/211472/02 | 2002 | USA;California | game owl |
| AY562988* | VI | chicken/U.S.(CA)/1083(Fontana)/72 | 1972 | USA | chicken |
| AY562989 | VI | dove/Italy/2736/00 | 2000 | Italy | dove |
| AY562990* | V | mixed species/U.S./Largo/71 | 1971 | USA | mixed species |
| AY562991‡ | I | chicken/N. Ireland/ Ulster/67 | 1967 | N.Ireland | chicken |
| AY741404* | IV | Herts/33 | 1933 | Holland | N.A. |
| AY845400‡ | II | LaSota | 1946 | China | N.A. |
| AY865652* | VII | Sterna/Astr/2755/2001 | 2001 | Russia | little tern |
| AY935489‡ | I | 01-1108 | 2001 | Australia | chicken |
| AY935490‡ | I | 02-1334 | 2002 | Australia | chicken |
| AY935491‡ | I | 98-1154 | 1998 | Australia | chicken |
| AY935492‡ | I | 98-1249 | 1998 | Australia | chicken |
| AY935493‡ | I | 98-1252 | 1998 | Australia | chicken |
| AY935494‡ | I | 99-0655 | 1999 | Australia | chicken |
| AY935495‡ | I | 99-0868hi | 1999 | Australia | chicken |
| AY935496‡ | I | 99-0868lo | 1999 | Australia | chicken |
| AY935497‡ | I | 99-1997PR-32 | 1999 | Australia | chicken |
| AY935498‡ | I | 99-1435 | 1999 | Australia | chicken |
| AY935499‡ | I | I-2 | N.A. | Australia | N.A. |
| AY935500‡ | I | I-2progenitor | N.A. | Australia | N.A. |
| DQ060053‡ | II | AQI-ND026 | N.A | China | N.A |
| DQ097394‡ | I | PHY-LMV42 | N.A. | Germany | N.A. |
| DQ485229* | VII | chicken/China/Guangxi7/2002 | 2002 | China;Guangxi | chicken |
| DQ485230 | VII | chicken/China/Guangxi9/2003 | 2003 | China;Guangxi | chicken |
| DQ485231* | VII | chicken/China/Guangxi11/2003 | 2003 | China;Guangxi | chicken |
| DQ486859* | VII | GM strain | N.A. | China | N.A. |
| DQ659677* | VII | NA-1 | N.A. | China | N.A. |
| DQ839397* | VII | KBNP-4152 | N.A. | Korea | N.A. |
| EF065682* | V | rAnhinga | N.A. | USA | N.A. |
| EF201805* | III | Mukteswar | N.A. | China;Lanzhou | N.A. |
| EU140955 | II | KBNP-C4152R2L vaccine strain | N.A. | Korea | N.A. |
| EU167540 | VII | SRZ03 | 2003 | China | chicken |
| EU289028‡ | II | VG/GA | N.A. | USA | N.A. |
| EU289029‡ | II | VG/GA | N.A. | USA | N.A. |
| EU293914* | IV | Italian | N.A. | China | N.A. |
| EU546165‡ | II | JL-1 | N.A. | China; Jilin | N.A. |
| FJ386392‡ | II | NDV01 | N.A. | China | chicken |
| FJ386393‡ | II | NDV02 | N.A. | China | chicken |
| FJ386394‡ | II | NDV03 | N.A. | China | chicken |
| FJ386395‡ | II | NDV04 | N.A. | China | chicken |
| FJ386396‡ | II | NDV05 | N.A. | China | chicken |
| FJ430159* | III | JS/7/05/Ch | 2005 | China | chicken |
| FJ430160* | III | JS/9/05/Go | 2005 | China | goose |
| Y18898‡ | II | clone 30 | N.A. | N.A. | N.A. |
| DQ097393** | - | strain DE-R49/99 | 1999 | N.A. | N.A. |

*Non-vaccine related sequences used in selection analyses.

‡ Vaccine-related sequences used in selection analyses.

**Class I aPMV-1 virus that was used as outgroup in phylogenetic analyses.

N.A. Not available.
