# Supplementary material for: The Effect of Vaccination on the Evolution and Population Dynamics of Avian Paramyxovirus-1
Source: PLoS Pathog. 2010 Apr 22;6(4):e1000872. doi: 10.1371/journal.ppat.1000872 (PMC2858710; doi:10.1371/journal.ppat.1000872)
Supplement: Table S2 — Full length F gene sequences used in BEAST analyses. (0.15 MB DOC) [file ppat.1000872.s002.doc]

**Table S2.** Full length F gene sequences used in BEAST analyses.

| Genbank  accession number | Genotype based on ML analysis | Description | Year | Host |
| --- | --- | --- | --- | --- |
| AY562991 | I | chicken/N. Ireland/Ulster/67 | 1967 | | chicken | | --- | |
| AY935489 | I | 01-1108 | 2001 | N.A |
| AY935490 | I | 02-1334 | 2002 | N.A |
| AY935491 | I | 98-1154 | 1998 | N.A |
| AY935492 | I | 98-1249 | 1998 | N.A |
| AY935493 | I | 98-1252 | 1998 | N.A |
| AY935494 | I | 99-0655 | 1999 | N.A |
| AY935495 | I | 99-0868hi | 1999 | N.A |
| AY935496 | I | 99-0868lo | 1999 | N.A |
| AY935497 | I | 99-1997PR-32 | 1999 | N.A |
| AY935498 | I | 99-1435 | 1999 | N.A |
| AY965077 | I | strain Anas/FarEast/3658/2002 | 2002 | Baikal teal |
| AY965078 | I | strain duck/FarEast/2686/2001 | 2001 | duck |
| AY965079 | I | strain duck/FarEast/2713/2001 | 2001 | duck |
| AY972101 | I | strain Anas/FarEast/3652/2002 | 2002 | Baikal teal |
| AY972102 | I | strain Anas/FarEast/3638/2002 | 2002 | Baikal teal |
| AY972103 | I | strain Duck/FarEast/2687/2001 | 2001 | duck |
| EF564816 | I | isolate red knot/US(NJ)/A101-1383/2001 | 2001 | red knot |
| EF564817 | I | isolate ruddy turnstone/US(DE)/492/2002 | 2002 | ruddy turnstone |
| EF564821 | I | isolate mallard/US(MD)/04-204/2004 | 2004 | mallard |
| FJ480814 | I | strain CK/CH/NX/1/06 | 2006 | chicken |
| FJ480815 | I | strain CK/CH/NX/2/06 | 2006 | chicken |
| FJ480816 | I | strain CK/CH/NX/3/06 | 2006 | chicken |
| FJ480817 | I | strain CK/CH/NX/4/06 | 2006 | chicken |
| AF309418 | II | B1 | 1947 | N.A |
| AF400614 | II | strain NDV-JL-1/97 | 1997 | N.A. |
| AF534997 | II | strain ZJ/2000 | 2000 | N.A |
| AY289000 | II | chicken/USA/Roakin/48 | 1948 | chicken |
| AY289002 | II | turkey/USA/VGGA/89 | 1989 | turkey |
| AY359876 | II | parrot/India/NDVCUL97 | 1997 | parrot |
| AY727883 | II | isolate 88T.00 | 2000 | flamingo |
| AY845400 | II | strain LaSota | 1946 | N.A |
| DQ023198 | II | strain FM1/03 | 2003 | semi-muscovy duck |
| DQ682436 | II | strain AH/1/04/Go | 2004 | goose |
| DQ682438 | II | strain JS/1/04/Go | 2004 | goose |
| DQ682439 | II | strain JS/1/05/Go | 2005 | goose |
| DQ682440 | II | strain SD/1/03/Go | 2003 | goose |
| DQ682441 | II | strain SD/1/04/Go | 2004 | goose |
| DQ682442 | II | strain SD/2/04/Go | 2004 | goose |
| DQ682443 | II | strain SD/3/04/Go | 2004 | goose |
| DQ682444 | II | strain SD/4/04/Go | 2004 | goose |
| DQ682446 | II | strain SD/6/04/Go | 2004 | goose |
| FJ480793 | II | strain CK/CH/HLJ/2/07 | 2007 | chicken |
| FJ480794 | II | strain CK/CH/HLJ/3/07 | 2007 | chicken |
| FJ480795 | II | strain CK/CH/HLJ/4/07 | 2007 | chicken |
| FJ480811 | II | strain CK/CH/LN/3/06 | 2006 | chicken |
| FJ480821 | II | strain Go/CH/FJ/1/06 | 2006 | goose |
| FJ480823 | II | strain Go/CH/HLJ/2/06 | 2006 | goose |
| FJ608340 | II | strain MQ/Liaoning/05 | 2005 | chicken |
| FJ608341 | II | strain TYQ/Shanxi/07 | 2007 | chicken |
| FJ608342 | II | strain KQ/Liaoning/06 | 2006 | chicken |
| FJ608344 | II | strain DF3Q/Beijing/08 | 2008 | chicken |
| AF358785 | VI | strain Ch/98-1 | 1998 | pigeon |
| AF456439 | VI | strain JS/2/98/Go | 1998 | Goose |
| AF458015 | VI | isolate ZhJ-3/97 | 1997 | chicken |
| AF458016 | VI | isolate ZhJ-2/86 | 1986 | chicken |
| AF458017 | VI | isolate Sh-2/98 | 1998 | chicken |
| AF458018 | VI | isolate Sh-1/97 | 1997 | chicken |
| AF458019 | VI | isolate XJ-3/97 | 1997 | chicken |
| AF458020 | VI | isolate XJ-1/91 | 1991 | chicken |
| AF458021 | VI | isolate JX-1/94 | 1994 | chicken |
| AJ880277 | VI | IT-227/82 | 1982 | pigeon |
| AY288996 | VI | pigeon/Italy/1166/00 | 2000 | pigeon |
| AY288997 | VI | chicken/Kenya/139/90 | 1990 | | chicken | | --- | |
| AY562988 | VI | chicken/U.S.(CA)/1083(Fontana)/72 | 1972 | chicken |
| AY734535 | VI | isolate Pigeon/Argentina/Tigre 6/99 | 1999 | Pigeon |
| AY734536 | VI | isolate Pigeon/Argentina/Capital 3/97 | 1997 | pigeon |
| EF520716 | VI | strain pigeon/NY/US/1984 | 1984 | pigeon |
| EU477188 | VI | strain dove/US/TX-B2580/2004 | 2004 | dove |
| EU477189 | VI | strain pigeon/US/RI166/2000 | 2004 | pigeon |
| EU477190 | VI | strain pigeon/US/TX3503/2004 | 2004 | pigeon |
| EU477191 | VI | strain Eurasian collared dove/US/TX3988/2004 | 2004 | Eurasian collared dove |
| EU477192 | VI | strain Eurasian collared dove/US/TX4156/2005 | 2005 | Eurasian collared dove |
| FJ480825 | VI | strain PG/CH/JS/1/05 | 2005 | pigeon |
| FJ480826 | VI | strain PG/CH/JS/1/06 | 2006 | pigeon |
| AF431744 | VII | strain ZJ1 | 2000 | Goose |
| AF473851 | VII | SF02 | 2002 | goose |
| AY288988 | VII | parakeet/Tanzania,Belgium,China/28710/93 | 1993 | parakeet |
| AY562985 | VII | cockatoo/Indonesia/14698/90 | 1990 | cockatoo |
| AY865652 | VII | strain Sterna/Astr/2755/2001 | 2001 | Little Tern |
| AY928933 | VII | isolate MK13/75 | 1996 | poultry |
| DQ485229 | VII | chicken/China/Guangxi7/2002 | 2002 | chicken |
| DQ485230 | VII | chicken/China/Guangxi9/2003 | 2003 | chicken |
| DQ485231 | VII | chicken/China/Guangxi11/2003 | 2003 | chicken |
| DQ485274 | VII | isolate dove/Guangxi15/2005 | 2005 | dove |
| EF128053 | VII | strain JS-3/06/wd | 2006 | wild duck |
| EF175145 | VII | isolate PX2/03 | 2003 | muscovy duck |
| EF521889 | VII | isolate mallard/China/HLJ-13-05 | 2005 | mallard |
| EU140947 | VII | strain SNU-0202 | 2000 | NA |
| EU140948 | VII | strain KBNP-4152 | 2004 | NA |
| EU140949 | VII | strain SNU-5070 | 2005 | NA |
| FJ480774 | VII | strain NDV/Buzzard/CH/HLJ009/06 | 2006 | rough-legged buzzard |
| FJ480775 | VII | strain NDV/Owl/CH/HLJ012/06 | 2006 | long-eared owl |
| FJ480777 | VII | strain NDV/MD/CH/HLJ028/06 | 2006 | Mandarin duck |
| FJ480778 | VII | strain NDV/WfG/CH/HLJ052/06 | 2006 | white-fronted goose |
| FJ480779 | VII | strain NDV/AG/CH/HLJ070/06 | 2006 | Japanese Sparrowhawk |
| FJ608351 | VII | strain YZCQ/Liaoning/08 | 2008 | chicken |
