# Supplementary material for: The Effect of Vaccination on the Evolution and Population Dynamics of Avian Paramyxovirus-1
Source: PLoS Pathog. 2010 Apr 22;6(4):e1000872. doi: 10.1371/journal.ppat.1000872 (PMC2858710; doi:10.1371/journal.ppat.1000872)
Supplement: Table S3 — The evolutionary rates and time to the most recent common ancestor (TMRCA) of each gene and concatenated genome based on GTR substitution model. (0.06 MB DOC) [file ppat.1000872.s003.doc]

**Table S3.** The evolutionary rates and time to the most recent common ancestor (TMRCA) of each gene and concatenated genome based on GTR substitution model.

| Gene | Clock model | Marginal likelihood | Evolutionary rate (×10^-3^ sub/site/yr) | Mean TMRCA^¶^  (95% HPD) | Bayes factor |
| --- | --- | --- | --- | --- | --- |
| NP | Strict | -6349.457 | 0.90 (0.73 - 1.07) | 1861 (1842 -1882) | 44.668 |
|  | UCED | -6304.789 | 0.98 (0.45 - 1.50) | 1868 (1713 - 1927) |  |
| P | Strict | -6240.857 | 1.28 (1.00 - 1.56) | 1886 (1867 - 1901) | 51.129 |
|  | UCED | -6189.728 | 1.56 (0.78 - 2.32) | 1884 (1750 - 1931) |  |
| M | Strict | -5142.709 | 1.02 (0.79 - 1.25) | 1878 (1857 - 1896) | 49.268 |
|  | UCED | -5093.441 | 1.17 (0.54 - 1.76) | 1872 (1737 - 1931) |  |
| F | Strict | -7810.551 | 1.09 (0.63 - 1.36) | 1881 (1838 - 1899) | 73.196 |
|  | UCED | -7737.355 | 1.35 (0.71 - 1.98) | 1891 (1777 - 1931) |  |
| HN | Strict | -8307.153 | 1.13 (0.93 - 1.35) | 1875 (1858 - 1890) | 88.586 |
|  | UCED | -8218.567 | 1.10 (0.51 - 1.68) | 1869 (1719 -1928) |  |
| L | Strict | -28416.82 | 0.96 (0.88 - 1.04) | 1878 (1871 - 1886) | 276.527 |
|  | UCED | -28140.293 | 1.02 (0.59 - 1.44) | 1878 (1764 -1924) |  |
| Concatenate | Strict | -62363.302 | 1.00 (0.94 - 1.06) | 1879 (1870 - 1881) | 572.505 |
|  | UCED | -61790.797 | 1.19 (0.50 – 1.74) | 1885 (1731 - 1929) |  |

Bayes Factor > 2.99 is considered significant.

^¶^The dates are presented as year.
