# Supplementary material for: The Effect of Vaccination on the Evolution and Population Dynamics of Avian Paramyxovirus-1
Source: PLoS Pathog. 2010 Apr 22;6(4):e1000872. doi: 10.1371/journal.ppat.1000872 (PMC2858710; doi:10.1371/journal.ppat.1000872)
Supplement: Table S4 — The evolutionary rates and time to the most recent common ancestor (TMRCA) of each gene and concatenated genome based on HKY substitution model, using both strict and relaxed (Uncorrelated Exponential) clock model. (0.06 MB DOC) [file ppat.1000872.s004.doc]

**Table S4.**

| Gene | Clock model | Marginal likelihood | Evolutionary rate (×10^-3^ sub/site/yr) | TMRCA^¶^ in year  (95% HPD) | Bayes factor |
| --- | --- | --- | --- | --- | --- |
| NP | Strict | -6371.01 | 0.91 (0.73 - 1.08) | 141 (122 - 161) | 44.904 |
|  | UCED | -6326.106 | 1.00 (0.48 - 1.49) | 153 (78 - 276) |  |
| P | Strict | -6254.804 | 1.25 (0.98 - 1.52) | 121 (104 - 139) | 50.654 |
|  | UCED | -6204.15 | 1.48 (0.75 - 2.21) | 143 (79 - 247) |  |
| M | Strict | -5155.239 | 1.02 (0.79 - 1.24) | 128 (110 - 149) | 49.939 |
|  | UCED | -5105.3 | 1.21 (0.63 - 1.76) | 133 (77 - 226) |  |
| F | Strict | -7822.793 | 0.91 (0.47 - 1.29) | 148 (109 - 215) | 73.280 |
|  | UCED | -7749.513 | 1.34 (0.76 - 1.94) | 131 (76 - 230) |  |
| HN | Strict | -8325.328 | 1.12 (0.85 - 1.36) | 132 (115 - 149) | 89.832 |
|  | UCED | -8235.496 | 1.05 (0.51 - 1.66) | 161 (78 - 276) |  |
| L | Strict | -28467.267 | 0.96 (0.88 - 1.05) | 127 (119 - 134) | 275.270 |
|  | UCED | -28191.997 | 1.05 (0.62 - 1.52) | 125 (79 - 186) |  |
| Concatenate | Strict | -62485.152 | 0.93 (0.84 - 1.02) | 129 (123 - 135) | 577.908 |
|  | UCED | -61907.244 | 1.00 (0.51 - 1.49) | 142 (84 - 230) |  |

Bayes Factor > 2.99 is considered significant.

^¶^ Year before 2008.
